# Detecting and Tracking Circulating Tumour DNA Copy Number Profiles during First Line Chemotherapy in Oesophagogastric Adenocarcinoma

**DOI:** 10.3390/cancers11050736

**Published:** 2019-05-27

**Authors:** Michael Davidson, Louise J. Barber, Andrew Woolston, Catherine Cafferkey, Sonia Mansukhani, Beatrice Griffiths, Sing-Yu Moorcraft, Isma Rana, Ruwaida Begum, Ioannis Assiotis, Nik Matthews, Sheela Rao, David Watkins, Ian Chau, David Cunningham, Naureen Starling, Marco Gerlinger

**Affiliations:** 1 Gastrointestinal and Lymphoma Unit, Royal Marsden NHS Foundation Trust, Sutton, London SM2 5PT, UK; Michael.Davidson@rmh.nhs.uk (M.D.); cafferkeycatherine@gmail.com (C.C.); SingYu.Moorcraft@rmh.nhs.uk (S.-Y.M.); Isma.Rana@rmh.nhs.uk (I.R.); Ruwaida.Begum@rmh.nhs.uk (R.B.); Sheela.Rao@rmh.nhs.uk (S.R.); David.Watkins@rmh.nhs.uk (D.W.); ian.chau@rmh.nhs.uk (I.C.); david.cunningham@rmh.nhs.uk (D.C.); Naureen.Starling@rmh.nhs.uk (N.S.); 2 Translational Oncogenomics Laboratory, Centre for Evolution and Cancer, Institute of Cancer Research, 237 Fulham Road, London SW3 6JB, UK; louise.barber@icr.ac.uk (L.J.B.); andrew.woolston@icr.ac.uk (A.W.); sonia.mansukhani@icr.ac.uk (S.M.); beatrice.griffiths@icr.ac.uk (B.G.); Ioannis.Assiotis@icr.ac.uk (I.A.); Nik.Matthews@icr.ac.uk (N.M.)

**Keywords:** oesophagogastric adenocarcinoma, circulating tumour DNA, somatic copy number aberration, liquid biopsy

## Abstract

DNA somatic copy number aberrations (SCNAs) are key drivers in oesophagogastric adenocarcinoma (OGA). Whether minimally invasive SCNA analysis of circulating tumour (ct)DNA can predict treatment outcomes and reveal how SCNAs evolve during chemotherapy is unknown. We investigated this by low-coverage whole genome sequencing (lcWGS) of ctDNA from 30 patients with advanced OGA prior to first-line chemotherapy and on progression. SCNA profiles were detectable pretreatment in 23/30 (76.7%) patients. The presence of liver metastases, primary tumour in situ, or of oesophageal or junctional tumour location predicted for a high ctDNA fraction. A low ctDNA concentration associated with significantly longer overall survival. Neither chromosomal instability metrics nor ploidy correlated with chemotherapy outcome. Chromosome 2q and 8p gains before treatment were associated with chemotherapy responses. lcWGS identified all amplifications found by prior targeted tumour tissue sequencing in cases with detectable ctDNA as well as finding additional changes. SCNA profiles changed during chemotherapy, indicating that cancer cell populations evolved during treatment; however, no recurrent SCNA changes were acquired at progression. Tracking the evolution of OGA cancer cell populations in ctDNA is feasible during chemotherapy. The observation of genetic evolution warrants investigation in larger series and with higher resolution techniques to reveal potential genetic predictors of response and drivers of chemotherapy resistance. The presence of liver metastasis is a potential biomarker for the selection of patients with high ctDNA content for such studies.

## 1. Introduction

Gastric and oesophageal cancers are challenging health issues, representing the third and sixth leading causes of global cancer mortality, respectively [1]. Advances have been made in the genetic characterisation and development of novel targeted agents for the adenocarcinoma histological subtype; however, the outlook for advanced disease remains poor with median overall survival not extending beyond 12 months in the majority of trials [2]. Recent large-scale sequencing projects have improved insights into the genomic landscape of the disease. The 2014 Cancer Genome Atlas (TCGA) analysis described four different subtypes of gastric cancer, with the most common chromosomal instability (CIN) subtype being characterised by chromosomal instability, aneuploidy, and, in many cases, focal amplification of receptor tyrosine kinases. The genomes of these cancers harbour multiple DNA somatic copy number alterations (SCNAs), defined as deviations in the number of whole chromosomes, chromosome arms, or fragments from the normal number of two copies per cell. With the exception of *p53* mutations, which occur in 70–80% of oesophagogastric adenocarcinomas (OGA) of the CIN subtype, mutations in cancer driver genes are relatively rare in these cancers, and SCNAs are considered the predominant type of genetic driver alterations [3,4]. Common SCNAs identified in CIN tumours in these landmark sequencing studies include amplifications of chromosomal regions harbouring genes encoding for receptor tyrosine kinases, or their ligands such as *ERBB2*, *EGFR*, and *VEGFA*, as well as those involved pathways regulating proliferation (*MYC*) and cell cycle (*CCNE1*, *CCND1*, and *CDK6*). These SCNAs have been implicated as key and, in the case of *ERBB2*/HER2, clinically actionable drivers in OGA [5,6].

The CIN subtype is common among gastric cancers arising proximally from the oesophagogastric junction or cardia [3] and in oesophageal adenocarcinomas [4]. The ‘genomically stable’ subtype is characterised by few SCNAs and associated with the diffuse histological subtype of gastric cancer that commonly arises more distally from the stomach body [3]. The incidence of noncardia gastric adenocarcinomas is declining in Western populations, whilst that of junctional and oesophageal adenocarcinomas is increasing [7]. These tumours are predominantly of the CIN subtype, and thus detection of SCNAs, in particular the clinically and biologically relevant driver events within these complex profiles, are important for the ongoing development of new biomarkers and therapies.

SCNAs have traditionally been analysed through microarray-based techniques, although more recently improved sensitivity for SCNA detection has been achieved through exome or whole genome sequencing (WGS). However, because of cost, long turnaround times, and intensive bioinformatics analysis requirements, such large-scale genomics analyses are often not feasible. Low-coverage WGS (lcWGS), using a coverage of only 0.1–0.5× (i.e., where only 10–50% of the genome is sequenced), has been shown to be sufficient for reliable detection of SCNAs with recent data showing superior SCNA calling compared to older array hybridisation-based standards [8]. Crucially, lcWGS can also be applied to analyse tumour-derived circulating free (cf)DNA extracted from the plasma of cancer patients [9]. Such liquid biopsies offer clear, practical advantages over conventional biopsies, including the minimally invasive nature of sample acquisition, relative ease of standardisation of sampling protocols, and the ability to obtain repeated samples over time. The latter is of particular interest, as changes in SCNA profiles over the course of treatment may shed light on response and resistance mechanisms to existing chemotherapy agents as well as to novel targeted agents and immunotherapies.

Intratumour heterogeneity is recognised as a major challenge in the delivery of effective molecular targeted treatment in OGA [10,11]. Copy number variation of molecular targets, as assessed in both tumour and cfDNA, has been shown to impact on therapeutic targeting of *ERBB2*, *FGFR*, and *EGFR*, with high level amplifications being associated with more favourable responses [12,13,14]. Application of targeted genomic sequencing to cfDNA analysis has been shown to allow the detection of mutations that are heterogeneous within OGA [15,16]. Such liquid biopsy techniques may also facilitate tracking of genetic profile changes over time, but this has not been applied to OGAs undergoing systemic therapy.

We applied lcWGS to cfDNA from 30 patients with advanced OGA to investigate whether SCNA analysis can predict responses to first-line chemotherapy and how these profiles may evolve during chemotherapy treatment.

## 2. Results

The clinical and pathological characteristics of the 30 included patients are summarized in Table 1. Extracted cfDNA concentrations from plasma samples taken at pretreatment baseline ranged from 1.37 to 74.04 ng/mL with a median of 8.88 ng/mL. With a minimum input quantity of 5 ng for lcWGS, sufficient cfDNA was available from all 30 patients. Univariate analysis showed that the presence of the primary tumour in situ was associated with a significantly increased cfDNA concentration (Table 2, 9.66 vs. 4.81 ng/mL, *p* = 0.0027, Mann–Whitney test). The cfDNA concentration was numerically higher in patients with liver metastases vs. those without liver metastases (10.09 vs. 6.80 ng/mL, *p* = 0.1306, Mann–Whitney test), but this was not significant. No other clinical or pathological parameters were associated with pretreatment cfDNA concentration. 

**Figure 1:**
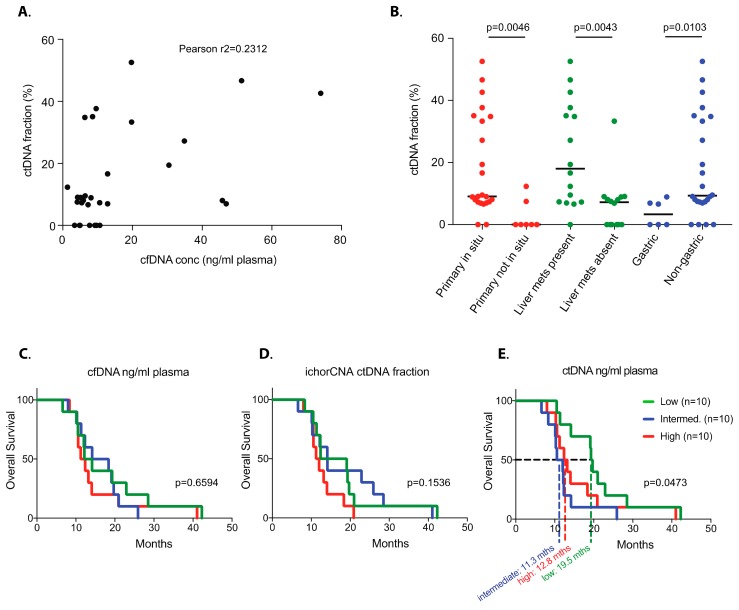
(**A**) No correlation between circulating free (cf)DNA concentration and the tumour-derived cfDNA fraction in 30 plasma samples from patients with treatment naïve metastatic gastro-oesophageal cancers. (**B**) Correlation between selected clinical features and circulating tumour (ct)DNA fraction (line denotes median; *p*-value Mann–Whitney test). (**C**) Kaplan–Meier survival analyses of pretreatment samples grouping by high/intermediate/low cfDNA yield ng/mL plasma, (**D**) ichorCNA ctDNA fraction, and (**E**) ctDNA concentration ng/mL plasma (*p*-values Log-rank (Mantel–Cox) test).

Sequencing was performed with 100 bp single-end reads and a target of 12 million reads per sample. The ichorCNA bioinformatics package [17] was used to reconstruct copy number profiles from sequencing data and to estimate the fraction of cfDNA that was derived from tumour cells (henceforth denoted as circulating tumour (ct)DNA content). Based on ichorCNA analysis, 7/30 cases (23.3%) had ctDNA content of zero, leaving 23 cases (76.7%) in which SCNA analysis could be performed. The seven cases with zero tumour content included all three tumours that were only locally advanced rather than metastatic in this cohort (Cases 2, 152, and 195). The other four (57.1%) cases with zero tumour content had metastatic disease involving only a single organ site (Cases 52, 66, 119, and 144). The ctDNA content showed a poor correlation with the total cfDNA concentration in the plasma (Figure 1A, Pearson correlation r^2^ = 0.2312), suggesting that the release of ctDNA from tumour cells and the total amount of cfDNA, which was a mix of DNA from malignant and nonmalignant cells, were largely independent from each other. The presence of the primary tumour in situ (9.1% vs. 0% median ctDNA content, *p* = 0.0046, Mann–Whitney test) and the presence of liver metastases (18.0% vs. 7.2% median ctDNA content, *p* = 0.0043, Mann–Whitney test) significantly correlated with higher ctDNA content (Table 2 and Figure 1B). A greater ctDNA content was also observed in oesophageal and junctional tumours compared to gastric tumours (9.3% vs. 3.3% median ctDNA content, *p* = 0.0103, Mann–Whitney test).

Taken together, copy number profiles could be analysed from cfDNA in 76.7% of cases and three distinct characteristics (primary tumour in situ, presence of liver metastases, and oesophageal/junctional primary tumour location) associated with high ctDNA content, with liver metastases showing the highest tumour fraction of 18% (median).

We next investigated whether any pretreatment cfDNA metrics correlated with overall survival (OS). Neither the total cfDNA concentration extracted from plasma (Figure 1C) nor the ctDNA content estimated by ichorCNA (Figure 1D) correlated with overall survival. However, the absolute ctDNA concentration in the plasma revealed a significant overall survival (OS) difference (Figure 1E). The third of patients with the lowest absolute ctDNA concentration (mean 0.09 ng/mL) had a median OS of 19.5 months, whereas those with intermediate (mean 0.92 ng/mL) and high (mean 10.12 ng/mL) absolute ctDNA concentrations had median OSs of 11.3 and 12.8 months, respectively.

**Figure 2:**
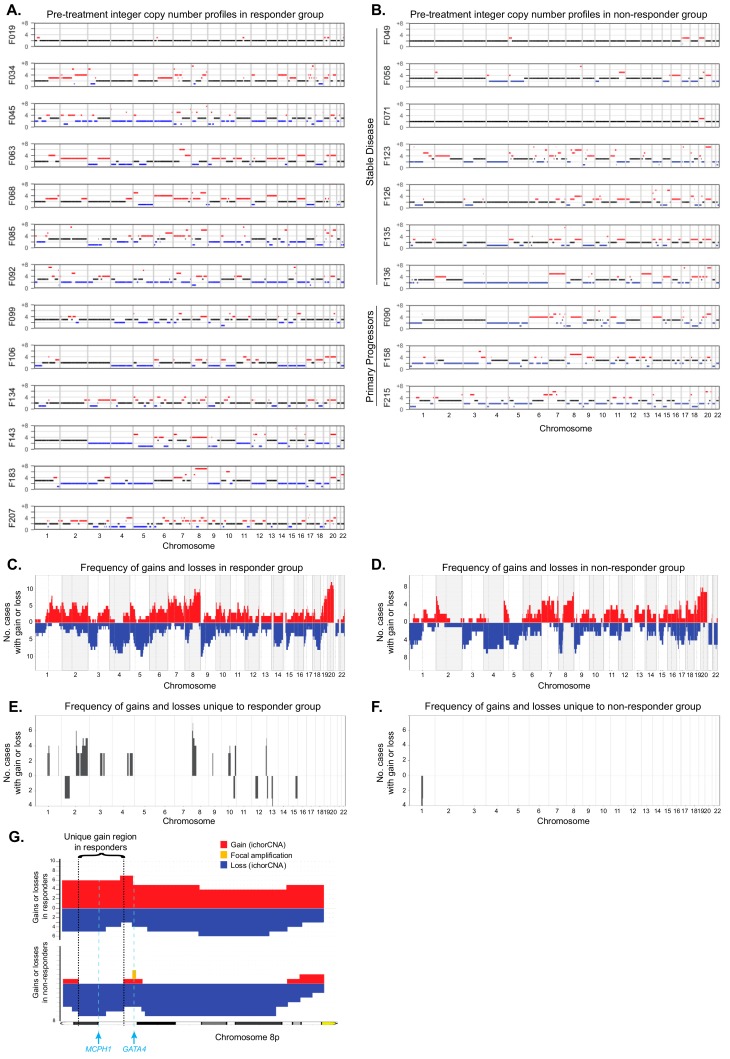
(**A**) Integer copy number profiles (500 kb bins) for pretreatment samples, grouped by subsequent response or (**B**) nonresponse to treatment. Red = gain, blue = loss, and black = ploidy. (**C**) Frequency plots showing the number of cases that show segment gains (red) or losses (blue) in the responder and (**D**) nonresponder groups. (**E**) Frequency plots showing segment gains and losses that are unique to the responder group or (**F**) nonresponder group. (**G**) Frequency of gain (red) and loss (blue) segments of chromosome 8p in the responder group (top) and nonresponder group (bottom). The most frequent region of unique 8p gain is indicated, bounded by dotted lines. The locations of *MCPH1* and *GATA4* are delineated with a blue dashed line. Two additional nonresponder cases showed focal amplifications (orange) of *GATA4*, which were identified with the 50 kb bin method but not the 500 kb ichorCNA analysis.

We next investigated whether any specific copy number aberrations or chromosomal instability metrics correlated with subsequent responses to chemotherapy (Figure 2A,B). The frequency of copy number gains or losses in 13 responders (based on best radiological response assessment with serial CT scans during treatment) (Figure 2C) was compared to those in 10 nonresponders who had stable or progressive disease as best response (Figure 2D). Frequency plots showed an overall similar appearance in both groups; however, several chromosomes showed alterations that were unique to the responders (Figure 2E) and not present in the nonresponder group (Figure 2F). Gains of chromosomes 2q and 8p were the most frequent (>1/3 of cases) unique aberrations observed only among responders (Figure 2E). A minimal consistent region of 28 Mb on Chr2q encompassing 182 genes was observed in five of 13 cases (34, 63, 68, 134, and 207). These 2q gains were in four cases a single copy number gain relative to ploidy. A 7.5 Mb minimal consistent region on Chr8p encompassing 17 genes (Appendix Table A1) was detected in six cases (34, 45, 68, 99, 143, and 183), four of which were multiple copies above ploidy. Of the uniquely gained genes, *MCPH1* (microcephalin) is notable as a key regulator of DNA damage response and a repressor of human telomerase reverse transcriptase function [18], and gains of *MCPH1* have been implicated in increased platinum sensitivity in nonsmall cell lung cancer [19] (Figure 2G). Chr8p also harbours *GATA4*, which is frequently gained or amplified in OGA [4,20], but this was located outside the unique region, as gains of *GATA4* were observed in both responders and nonresponders (Figure 2G). Other uniquely altered regions were less frequent and, hence, difficult to assess (Figure 2E). In contrast, only a single loss of a 12 Mb minimal consistent region encompassing 117 genes on Chr1p in four cases (123, 126, 90, and 158) was unique to the nonresponder group (Figure 2F).

**Figure 3:**
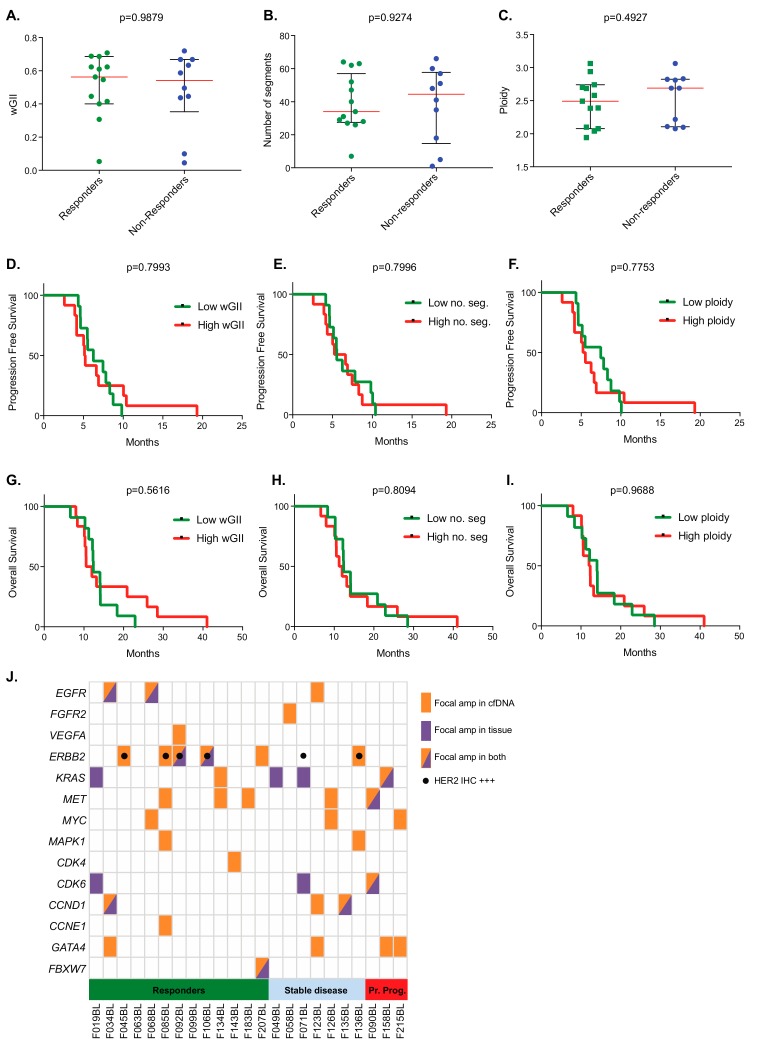
(**A**) Association of pretreatment chromosomal instability (CIN) metrics with subsequent treatment response by comparing analysis of genomic change relative to ploidy using weighted genomic instability index (wGII), (**B**) nonploidy segment number, and (**C**) ploidy between responder and nonresponder groups (line denotes median and interquartile range; *p*-value Mann–Whitney test). (**D**) Kaplan–Meier progression free survival analyses grouping by high/low wGII, (**E**) nonploidy segment number, and (**F**) ploidy. (**G**) Kaplan–Meier overall survival analyses grouping by high/low wGII, (**H**) nonploidy segment number, and (**I**) ploidy. (**J**) Heatmap showing focal gene amplifications (50 kb bins) detected by cfDNA lcWGS at pretreatment (orange) or by archival target sequencing (purple) in each case. Black dots indicate cases classed as HER2+ by immunohistochemistry. Green = responder group, blue *=* stable group, and red = primary progressor group.

Chromosomal instability (CIN) has been associated with poor outcomes and treatment responses in several cancer types [21,22]. We hence assessed whether CIN metrics including the weighted genomic instability index (wGII) [23,24] (Figure 3A), the number of gained or lost chromosomal segments (Figure 3B), or ploidy (Figure 3C), associated with responses or could predict survival in our cohort. None of these metrics showed a significant difference in responders vs. nonresponders or an association with progression-free (Figure 3D–F) or overall survival (Figure 3G–I). Taken together, the presence of Chr2q and 8p gains in pretreatment ctDNA showed an association with chemotherapy responses. In contrast, we could not identify a role of CIN metrics to predict patient outcomes in OGA.

The ichorCNA analysis divides chromosomes into 500 kb large bins to robustly assess the copy number state of these segments. Focal genomic amplifications are often narrow [4] (down to a few dozen kbps) and may have been overlooked as a consequence. Therefore, to further interrogate whether focal amplifications could be detected in the lcWGS data, we applied a 50 kbp bin approach [25]. This revealed narrow high-level amplifications of several OGA driver genes [3,4] (Figure 3J). Any of the high-level amplifications (*EGFR*, *ERBB2*, *KRAS*, *MET*, *MYC*, *MAPK1/ERK2*, *CCND1*, and *GATA4*) that were observed in two or more cases were detected in both responders and in nonresponders. Several others were only observed once and were, hence, too rare to draw any conclusions. Thus, high-level amplifications detected pretreatment were not associated with chemotherapy responses.

As part of the FOrMAT clinical trial, archival formalin-fixed paraffin-embedded diagnostic or resection samples were sequenced with a custom panel, which targeted 46 genes that had prognostic or predictive significance or were potential targets in existing or upcoming clinical trials [26]. Amplifications of *EGFR*, *CCND1*, *CDK6*, *MET*, *ERBB2*, *KRAS*, and *FBXW7* had been identified in tissue samples from 11 cases (19, 34, 49, 68, 71, 90, 92, 106, 135, 158, and 207). No amplifications were observed in nine cases, and archival target sequencing failed in three cases (45, 58, and 123). cfDNA lcWGS of pretreatment plasma reidentified all gene amplifications found by archival tumour sequencing in eight cases (Figure 3J). Compared to tissue sequencing, ctDNA analysis could not detect *CDK6* and/or *KRAS* amplifications in three cases that had low ctDNA content (Case 19: 9.1%; Case 49: 7.3%; and Case 71: 8.1%). Importantly, in seven cases, cfDNA lcWGS identified additional amplifications of genes that were included in the FOrMAT sequencing panel but for which no amplification was detected in the archival tissue analysis: Case 85 (*MET* and *ERBB2* amplification in plasma), Case 126 (*MET*), Case 134 (*MET*, *KRAS*), Case 136 (*ERBB2*), Case 143 (*CDK4*), Case 183 (*MET*), and case 207 (*ERBB2*). In addition, cfDNA sequencing identified 11 amplifications (in nine cases) of genes that were not covered by the FOrMAT panel including *GATA4*, *VEGFA*, and *MYC*.

Of six cases (45, 71, 85, 92, 106, and 136) that had been classified as HER2-positive based on standard immunohistochemistry testing of archival tissue, cfDNA sequencing detected *ERBB2* amplifications in five cases. Archival tissue sequencing had identified *ERBB2* amplifications in only two of five successfully sequenced cases (Figure 3J). In one case (71), immunohistochemical (IHC) analysis of archival tissue had identified HER2 positivity, but no amplification was detected by either archival tumour sequencing or cfDNA lcWGS. Three of the *ERBB2* amplified cases (85, 92, and 136) had concurrent amplifications in *MAPK1*, *MET*, or *VEGFA* in the cfDNA (Figure 3J).

**Figure 4:**
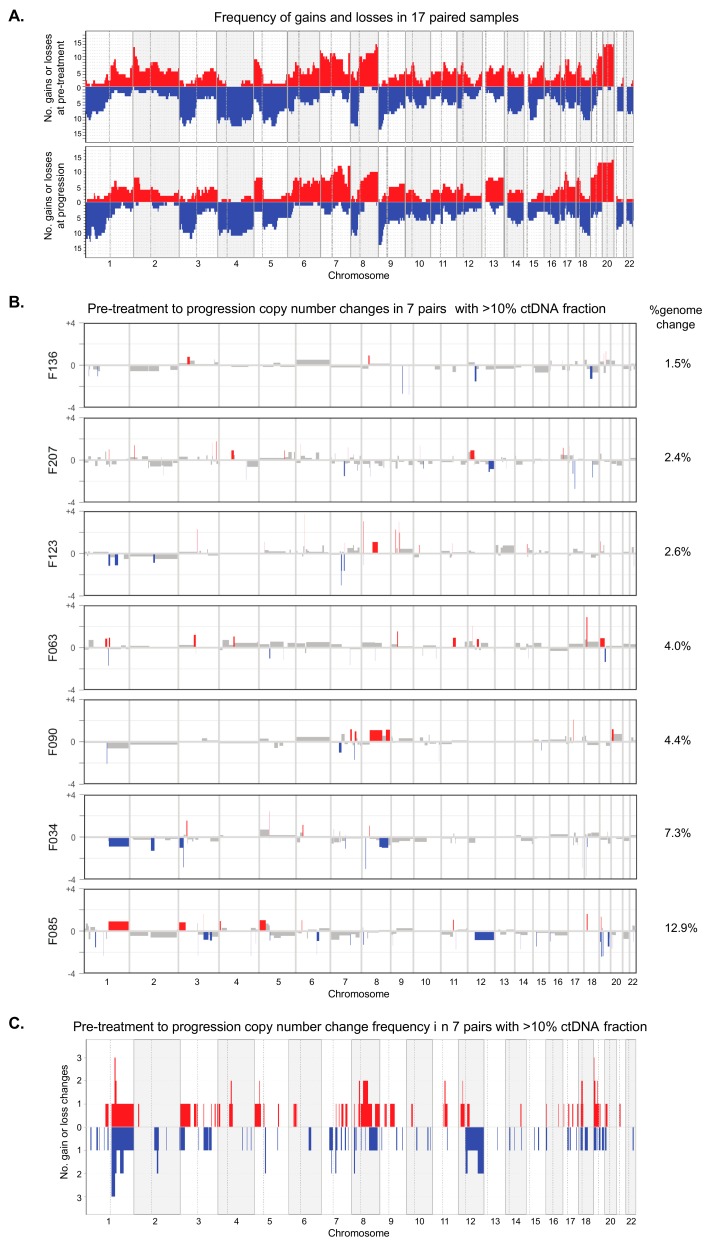
(**A**) Frequency plots showing the number of cases (*n* = 17) that show segment gains (red) or losses (blue) at pretreatment (top) and at progression (bottom). (**B**) For 7 pairs where both samples had >10% ctDNA fractions, comparative plots show absolute copy number gains and losses at progression relative to pretreatment, ordered by the extent of genomic change. The percent genomic change for each sample is indicated to the right of each plot. Red *=* gain, blue *=* loss, and black *=* no change. A minimum of 0.8 copy number change was required to score a gain or a loss. (**C**) Frequency plot showing the number of cases (*n* = 7) that show segment gains (red) or losses (blue) at progression relative to pretreatment.

**Figure A1:**
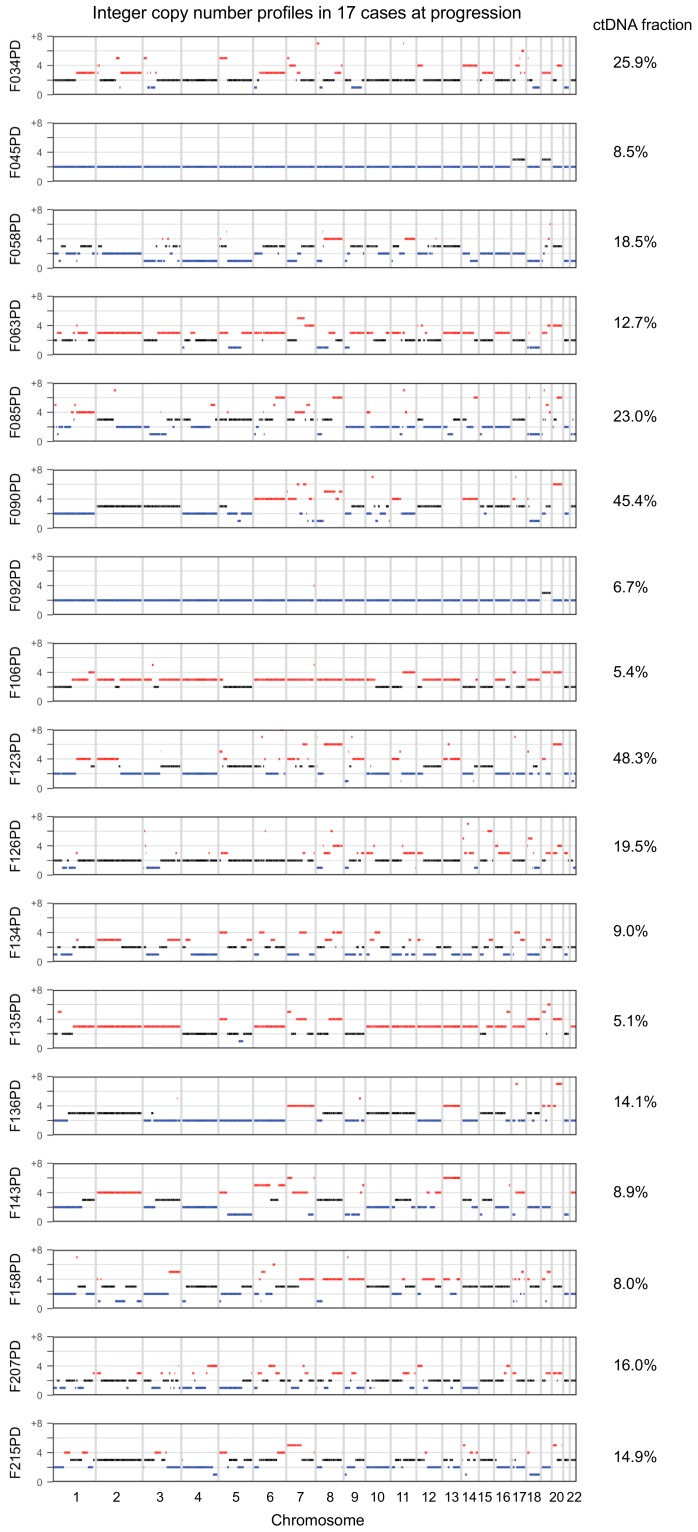
Integer copy number profiles for the 17 paired non-zero ctDNA cases at progression. ichorCNA ctDNA fraction is indicated for each sample.

lcWGS was applied to cfDNA collected at the time of radiological progression, during or after first line treatment, from 20 patients that had detectable ctDNA pretreatment profiles and had a post-treatment sample available. Twelve of these had an initial radiological response with subsequent disease progression (primary responders). Eight showed stable disease or primary progression during chemotherapy (primary nonresponders). In the primary responder group, the ichorCNA ctDNA fraction at progression was significantly lower than at pretreatment (17% vs. 7.6%, *p* = 0.02, Table 3), whereas no significant change was observed in the primary nonresponder group. Only three out of twenty samples taken at progression had a ctDNA content of zero (Cases 68, 99, and 183), showing that ctDNA remained detectable in the majority of tumours. The copy number profiles of the remaining 17 cases (Appendix Figure A1) were assessed for changes over the course of chemotherapy treatment (Figure 4A). Using the 50 kb bin approach, all focal amplifications present before treatment were reidentified at progression. No new focal amplifications were identified at progression.

In a second approach, we subtracted the pretreatment absolute copy number (generated with ichorCNA) from the absolute copy number in the matched progression sample to assess which chromosomes changed through chemotherapy. To avoid artefacts from differences in tumour content, this pairwise comparison was only performed in seven cases where tumour content was similar and above 10% at both pre-treatment and progression. Only changes of the integer copy number value exceeding +/−0.8 were considered; this was to enrich for new aneuploidies that had likely occurred in the majority of cells in the tumour and to avoid overinterpretation of changes in small subclones. The SCNA profiles were overall similar before treatment and at progression, but multiple individual segmental and arm-level changes were observed (Figure 4B). The fraction of the genome that changed (defined as the percent of the total genomic length that changed) was higher in responders (median: 5.65%, *n* = 4) than in nonresponders (median: 2.6%, *n* = 3, Figure 4B), but this was not statistically significant. Individual cases showed new gains or losses in multiple chromosomes. However, most of the genomic regions that changed between pretreatment and progression were only observed in a single case, and no large regions were changed in more than two cases (Figure 4C).

## 3. Discussion

Through use of liquid biopsy, we successfully analysed the SCNA profiles of 76.7% of 30 advanced OGAs. Serial analyses before and after first line chemotherapy were feasible in 85% of cases (17/20) that had detectable ctDNA prior to treatment. This demonstrates proof of concept that lcWGS of cfDNA can reveal genome-wide SCNA profiles in the majority of patients with advanced OGA to, for example, investigate novel prognostic or predictive biomarkers.

We identified several clinical characteristics that should support the selection of patients with predictably higher cfDNA analysis success rates in future studies: the presence of liver metastases was associated with the highest ctDNA concentrations, whilst the ctDNA concentration was also significantly higher if the primary tumour was in situ. This may be the result of more aggressive tumours presenting with synchronous metastatic disease at baseline compared to those with metachronous metastases following resection. All seven cases with zero ctDNA pretreatment either only had locally advanced disease or low metastatic burden. The use of such biomarkers to select OGA patients who are suitable for cfDNA sequencing may allow prioritizing these for liquid biopsy-based genotyping over sequencing of OGA tumour tissue, which has had moderate reported success rates because of technical challenges such as frequent low-tumour content in endoscopic biopsies [26,27]. With readily assessable clinical characteristics to identify suitable patients, cfDNA analysis could become the method of choice to assess amplifications for molecular stratification and, particularly, to longitudinally investigate SCNA evolution.

Neither pretreatment total cfDNA concentration nor ctDNA tumour content correlated with survival; however, a low absolute plasma ctDNA concentration was significantly associated with better OS. A previous gastric cancer case series described an association between baseline cfDNA and both relapse risk and adverse prognosis in the advanced disease setting [28]; however, larger studies are needed to validate the clinical utility of such metrics for optimisation of treatment and surveillance strategies [29].

High chromosomal instability (CIN) has been linked to poorer prognosis and drug sensitivity across a range of cancer types and to drug resistance in vitro [22,30]. Application of several CIN metrics could not identify a correlation with chemotherapy response or survival in our cohort. This could indicate that CIN metrics may perform less well when generated from ctDNA, as this samples a summative copy number profile of the entire cancer population. Alternatively, these metrics may only weakly correlate with aggressiveness and treatment sensitivity and specific genetic aberrations, acquired as a consequence of CIN, which may be more relevant in determining the response and outcome of individual tumours. Although studies of larger cohorts may be able to reveal an association in the future, our results suggest that analysis of these CIN metrics in ctDNA is unlikely to be useful to predict individual patient outcomes in unselected patients undergoing first line chemotherapy.

For patients with evaluable ctDNA, multiple SCNAs could be identified in genes that were currently clinically relevant or might become relevant to future practice. In samples with detectable ctDNA, we identified all amplifications that had been found by previous targeted sequencing of matched FFPE tissue samples [26]. In seven cases, lcWGS found an additional nine focal amplifications in genes that had been analysed by targeted sequencing in tissue (*ERBB2*, *MET*, *KRAS*, and *CDK4*) and where no amplification had been called. In three cases where tumour tissue sequencing failed, amplifications in *ERBB2*, *FGFR2*, *EGFR*, and *CCND1* were identified in ctDNA. Furthermore, lcWGS revealed multiple additional amplifications of potentially targetable driver genes such as *VEGFA*, highlighting the advantage of whole genome approaches over predetermined targeted sequencing gene sets.

Concurrent pretreatment amplifications of *MAPK1*, *MET*, or *VEGFA* with *ERBB2* were seen in 3/6 HER2-positive cases. These may potentially influence variability of outcomes to HER2-targeted therapy, as amplifications of *MET* and *MAPK1* have previously been implicated in trastuzumab resistance [31,32]. However, the limited numbers in this cohort precluded meaningful survival analyses.

Comparison of pretreatment SCNA profiles revealed gains of chromosomes 2q and 8p in cases that subsequently responded to treatment, and these gains were absent in nonresponders. These need to be investigated in larger cohorts to assess their potential role as predictive biomarkers. The uniquely gained region on chromosome 8p harbours the DNA damage regulator *MCPH1*, which has been suggested to increase sensitivity to platinum chemotherapy [19]. This is, therefore, a candidate gene for further investigation. Identifying predictive biomarkers of chemotherapy response is an unmet need. To date, the most extensive study of genetic predictors of therapy response using targeted sequencing of tumour tissue in advanced OGA failed to identify any biomarkers of response to platinum-based chemotherapy [27].

Both ctDNA detection and lcWGS were possible from plasma samples taken at the timepoint of progression on first line chemotherapy, with 17/20 (85%) cases having detectable ctDNA. SCNA profiles were relatively stable between the pretreatment and progression samples, but segmental and whole chromosomal arm changes were detected in seven cases where pair-wise comparisons were quantifiable. As it was unlikely that multiple subclones within a cancer would all gain or lose the same chromosomal regions, these copy number changes suggested that there had been major shifts in the clonal composition of the tumour cell populations, where one or a few subclones became dominant whereas others had been lost. lcWGS may, therefore, be a useful technology for the investigation of resistance landscapes in larger cohorts. The lack of recurrent copy number change events at progression in this study may be a result of the small evaluable cohort, but equally it is feasible that chemotherapy resistance may be driven by point mutations. Use of a higher resolution technique that will allow the combined analysis of SCNA and mutations (such as whole exome cfDNA sequencing) may be warranted, with patient selection based on the presence of liver metastases to maximise successful sequencing rates and cost efficiency. Longitudinal cfDNA analysis has become a favoured method to interrogate resistance mechanisms during treatment, such as the tracking of known oncogenic *RAS* mutations in colorectal cancer [33]. Dynamic cfDNA testing should be equally applicable to monitor resistance to therapy in OGA.

The potential clinical application for this technique lies in the feasibility of biomarker stratification on the basis of lcWGS cfDNA sequencing, circumventing some of the limitations related to tumour heterogeneity in OGA [13]. Furthermore, sequential lcWGS of cfDNA is a low-cost method for continuing to investigate genetic changes associated with chemotherapy response in larger series or for early detection of resistance mechanisms to novel agents in clinical trials. Preliminary proof of concept for the use of longitudinal cfDNA analyses to predict response and resistance to HER2-targeting treatment has already been described [34]. *ERBB2* copy number alterations detected by targeted sequencing were found to be associated with both innate and acquired trastuzumab resistance. Additionally, mutations in genes, including *PIK3CA*, *ERBB2*, and *ERBB4*, were also associated with resistance, highlighting the benefit of combined mutation identification and SCNA analysis in interrogating drug resistance mechanisms. Detection of relevant gene amplifications in cfDNA has been already shown to be clinically important for patient selection and therapeutic targeting of *FGFR* in gastric cancer [13]. However, plasma contains multiple components in addition to cfDNA that could also be utilised to realise the full potential of the liquid biopsy. Promising techniques under investigation in OGA include the enumeration and characterization of circulating tumour cells (CTCs), which have been associated with both prognosis [35] and treatment response [36]. In prostate cancer, mRNA extracted from CTCs has been used to identify splice variants of the androgen receptor that are prognostic for taxane therapy [37]. Furthermore, CTCs from small cell lung cancer have been successfully cultured ex vivo in order to screen for targeted therapy sensitivity and relevant biomarkers [38,39]. As an alternative to CTCs and cell-free nucleic acids, exosomes may also provide a means for tumour profiling, including in OGA [40].

As novel targeted and immune-modulating therapies are introduced into clinical management of OGA, there will be a need for stratification of patients in order to guide personalised treatment. The use of genome-wide analysis to interrogate key driver events and genomic evolution over time will be important in refining the effective biomarker stratification of such treatments moving forward. It is possible that a combination of lcWGS cfDNA sequencing with CTC or exosome analyses will facilitate maximal clinical utility to be gained from liquid biopsy approaches in order to guide treatment decisions. Ultimately this may support precision medicine in both trial and routine clinical practice settings by avoiding the cost, delay, and clinical complications of repeated invasive biopsy procedures.

## 4. Methods

### 4.1. Trial Design and Sample Collection

The FOrMAT (Feasibility of a Molecular Characterisation Approach to Treatment, Chief Investigator: N Starling ClinicalTrials.gov NCT02112357) study enrolled patients with advanced gastrointestinal malignancies treated at the Royal Marsden from February 2014 to November 2015 [26]. The trial was approved by the UK National Ethics Committee (approval number: 13/LO/1274RM), and all patients provided written informed consent. As part of the tissue collection component of the trial, blood samples were obtained at trial entry and at the timepoint of response assessment CT scans during treatment. The trial recruited 71 advanced OGA cancer patients in total. The clinical trial database was interrogated to identify 30 patients with a diagnosis of locally advanced inoperable or metastatic OGA. These patients had undergone baseline research blood sampling prior to commencement of treatment, and had sequential bloods spanning at least the full course of comparable first-line systemic chemotherapy. This consisted of a platinum/fluoropyrimidine doublet in all cases, plus or minus anthracycline or, in the case of *ERBB2*-positive tumours, trastuzumab. cfDNA was extracted from plasma samples taken at a baseline pretreatment timepoint for all patients. To assess the evolution of SCNA profiles through treatment, lcWGS was additionally performed on cfDNA collected at the time of radiological progression during or after first line platinum and 5FU-based combination chemotherapy from 20 patients that had detectable ctDNA pretreatment profiles and had a post-treatment sample available.

### 4.2. Circulating Free (cf)DNA Extraction and Quantification

Plasma was separated within 2 h of blood draw and frozen at −80 °C. The QIAamp Circulating Nucleic Acid Kit (Qiagen, Hilden, Germany) was used to isolate cfDNA from 3–4 mL plasma according to manufacturer’s instructions. cfDNA within a size range of 100 to 700 bp was quantified using a Bioanalyzer High Sensitivity chip (Agilent, Santa Clara, CA, USA), encompassing the predominant three cfDNA fragment peaks [41].

### 4.3. Low-Coverage Whole Genome Sequencing (lcWGS)

For the majority of cases, 10 ng of input DNA was used for sequencing, although 5 ng was used in some cases with limited yield [42]. Libraries were prepared using the NEBNext Ultra DNA Library Prep kit (NEB, Ipswich, MA, USA), which were pooled and sequenced on an Illumina HiSeq2500 in Rapid mode single read 100 bp.

### 4.4. Somatic Copy Number Aberration (SCNA) Analysis

Sequencing reads were aligned to the human reference genome (hg19) using Bowtie (v1.2.9) [43], and resultant bam files were deduplicated using Picard MarkDuplicates (http://picard.sourceforge.net; v.2.1.0). Reads were subsequently assigned to nonoverlapping 500 kb bins and normalized to correct for GC content and mappability bias using the HMMcopy suite (http://compbio.bccrc.ca/software/hmmcopy/) [44]. IchorCNA [17] was used to quantify tumour fraction in cfDNA from lcWGS without prior knowledge of somatic single nucleotide variants (SSNVs) or SCNAs present in the primary tumour sample. IchorCNA segmented data were normalised using the best-fit tumour content and ploidy solution in order to compare samples. To compare multiple samples, data were uniformally segmented using interpolate.pcf, which was part of the copynumber package in R (http://bioconductor.org/packages/copynumber/) [45]. Cohort frequency plots were generated using the copynumber plotFreq function. Seg files were viewed as a heat map using the Integrated Genome Viewer (IGV) software (Broad Institute, Cambridge, MA, USA; v.2.3.97), allowing comparison of genomic SCNA profiles across multiple samples with the ability to zoom in to areas of interest in order to investigate genes located within this genomic region [46]. Focal SCNAs were identified by assigning mapped reads to 50 kb bins using the method described by Baslan [25]. SCNAs were assessed in IGV by two independent observers and recorded for all patients.

### 4.5. Survival Analyses by Pre-Treatment Circulating DNA Metrics

Tertile survival analyses were undertaken according to three circulating DNA metrics: (1) total cfDNA concentration extracted from plasma, (2) ctDNA content estimated by ichorCNA, and (3) absolute ctDNA concentration in the plasma, calculated by multiplying the total cfDNA concentration with the ichorCNA ctDNA content. In each case, the 30 samples were classified into ‘low’, ‘medium’, and ‘high’ tertiles for each metric, and the overall survival trend was analysed using the log-rank method.

### 4.6. Data Availability

Sequence reads have been deposited in the European Genome Phenome Archive (ID: submission ongoing—will be updated as soon as ID assigned).

## 5. Conclusions

SCNA profiles were successfully analysed through the use of lcWGS applied to cfDNA extracted from pretreatment baseline plasma samples in 23/30 (76.7%) cases. The presence of liver metastases, primary tumour in situ, and oesophageal or junctional primary tumour site were associated with higher pretreatment ctDNA content, and a lower baseline ctDNA concentration was associated with subsequent improved overall survival. Concordance was noted with prior targeted tumour sequencing results. Additionally, lcWGS revealed additional amplifications of potentially targetable driver genes, highlighting the advantage of whole genome approaches over predetermined targeted sequencing gene sets. ctDNA detection and lcWGS were possible from plasma samples taken at the timepoint of progression on first line chemotherapy, with SCNA profiles successfully analysed in 17/20 (85%) cases. Although SCNA profiles were relatively stable between pretreatment and progression, segmental and whole chromosomal arm changes were detected in seven cases where pair-wise comparison was quantifiable. Such shifts in the clonal composition of tumour cell populations during chemotherapy warrant further investigation as a possible dynamic means of investigating resistance landscapes in OGA.

## Figures and Tables

**Table 1:** Clinical characteristics of included patients.

Histopathological Variable		
**Number of Cases:**		30
Anatomic site of primary:	Gastric	6 (20%)
	OGJ/oesophageal	24 (80%)
Histological subtype:	Intestinal	28 (93%)
	Diffuse	2 (7%)
Clinical stage at presentation:	Locally advanced	3 (10%)
	Metastatic	27 (90%)
HER2 status *:	Positive	6 (20%)
	Negative	24 (80%)
First line chemotherapy:	Platinum/fluoropyrimidine doublet	9 (30%)
	Doublet+ anthracycline	15 (50%)
	Doublet+ trastuzumab	6 (20%)
Metastatic sites: Liver	Yes	16 (53%)
	No	14 (47%)
Peritoneal	Yes	6 (20%)
	No	24 (80%)
Lung	Yes	8 (27%)
	No	22 (73%)
Number of metastatic organ sites:	0–1	22 (73%)
	≥2	8 (27%)
Primary tumour in situ:	Yes	23 (77%)
	No	7 (23%)
CA19-9 secretor:	Yes	15 (50%)
	No	15 (50%)

* defined as HER2 immunohistochemical (IHC) +++ on baseline diagnostic specimen from patient clinical records; OGJ—oesophagogastric junction.

**Table 2:** Correlation of cfDNA concentration, median ichorCNA ctDNA fraction, and ctDNA concentration with clinical and laboratory variables (*p*-values Mann–Whitney test).

Histopathological Variable		*n*	Median cfDNA Concentration (ng/mL Plasma)	*p*-Value	Median ctDNA Fraction (%)	*p*-Value	Median ctDNA Concentration (ng/mL Plasma)	*p*-Value
Primary tumour in situ	Yes	23	9.66	0.0027	9.10	0.0046	2.14	<0.0001
	No	7	4.81		0.00		0.00	
Liver metastases present	Yes	16	10.09	0.1306	18.01	0.0043	2.18	0.0099
	No	14	6.80		7.23		0.35	
Primary tumour anatomic site	Gastric	6	8.65	0.8996	3.33	0.0103	0.24	0.1401
	Nongastric	24	9.05		9.31		0.84	
No. of metastatic organ sites	0–1	22	8.31	0.5042	7.77	0.1528	0.47	0.9814
	≥2	8	1.22		14.47		0.58	
HER2 status	Positive	6	11.22	0.3739	8.81	0.4595	2.25	0.1713
	Negative	24	8.32		8.22		0.47	
CA19-9 secretion	Yes	15	9.21	0.9999	8.10	0.5640	0.61	0.7733
	No	15	8.54		9.02		0.78	

**Table 3:** Comparison of ichorCNA estimated ctDNA fraction at pretreatment and progression of first line chemotherapy (*p*-values Mann–Whitney test).

Paired Pretreatment and Progression Cases		*n*	Median ctDNA Fraction (%)	*p*-Value
All paired cases	Pretreatment	20	15.18	0.1567
	Progression	20	8.72	
Initial radiological response followed by progression to chemotherapy: ‘primary responders’	Pretreatment	12	17.00	0.0200
	Progression	12	7.59	
Stable disease or primary radiological progression to chemotherapy: ‘primary nonresponders’	Pretreatment	8	11.27	0.7984
	Progression	8	13.58	

## Appendix A

**Table A1:** Genes in frequently gained region of chromosome 8p in responders.

*CSMD1*
*LOC100287015*
*MCPH1*
*ANGPT2*
*CLDN23*
*MFHAS1*
*ERI1*
*MIR4660*
*PPP1R3B*
*LOC157273*
*TNKS*
*MIR597*
*LINC00599*
*MIR124-1*
*MSRA*
*PRSS55*
*RP1L1*
